# Selective MicroRNA-Offset RNA Expression in Human Embryonic Stem Cells

**DOI:** 10.1371/journal.pone.0116668

**Published:** 2015-03-30

**Authors:** Suvi Asikainen, Liisa Heikkinen, Juuso Juhila, Frida Holm, Jere Weltner, Ras Trokovic, Milla Mikkola, Sanna Toivonen, Diego Balboa, Riina Lampela, Katherine Icay, Timo Tuuri, Timo Otonkoski, Garry Wong, Outi Hovatta

**Affiliations:** 1 Department of Clinical Science, Intervention and Technology, Karolinska Institutet, 17177, Stockholm, Sweden; 2 Research Programs Unit, Molecular Neurology and Biomedicum Stem Cell Centre, University of Helsinki, 00014, Helsinki, Finland; 3 A.I. Virtanen Institute, University of Eastern Finland, 70211, Kuopio, Finland; 4 Department of Biological and Environmental Science, University of Jyvaskyla, 40014, Jyvaskyla, Finland; 5 Children’s Hospital, Helsinki University Central Hospital, 00029, Helsinki, Finland; 6 Faculty of Health Sciences, University of Macau, Taipa, Macau S.A.R., China; IRCCS-Policlinico San Donato, ITALY

## Abstract

Small RNA molecules, including microRNAs (miRNAs), play critical roles in regulating pluripotency, proliferation and differentiation of embryonic stem cells. miRNA-offset RNAs (moRNAs) are similar in length to miRNAs, align to miRNA precursor (pre-miRNA) loci and are therefore believed to derive from processing of the pre-miRNA hairpin sequence. Recent next generation sequencing (NGS) studies have reported the presence of moRNAs in human neurons and cancer cells and in several tissues in mouse, including pluripotent stem cells. In order to gain additional knowledge about human moRNAs and their putative development-related expression, we applied NGS of small RNAs in human embryonic stem cells (hESCs) and fibroblasts. We found that certain moRNA isoforms are notably expressed in hESCs from loci coding for stem cell-selective or cancer-related miRNA clusters. In contrast, we observed only sparse moRNAs in fibroblasts. Consistent with earlier findings, most of the observed moRNAs derived from conserved loci and their expression did not appear to correlate with the expression of the adjacent miRNAs. We provide here the first report of moRNAs in hESCs, and their expression profile in comparison to fibroblasts. Moreover, we expand the repertoire of hESC miRNAs. These findings provide an expansion on the known repertoire of small non-coding RNA contents in hESCs.

## Introduction

Human embryonic stem cells (hESC) are pluripotent cells derived from the inner cell mass of blastocyst stage embryos, which can be indefinitely maintained in culture [1–3]. The pluripotency, proliferation, and differentiation of hESCs are influenced by transcription factors that mediate their actions in concert with miRNAs, small endogenous RNAs processed by RNAse III endonucleases Dicer and Drosha [4–8]. With the ability of a single miRNA to regulate hundreds of genes [9], stem cell miRNAs are postulated to fine-tune developmental gene expression programs and provide robustness (and plasticity) to cell fate determinations [10–12]. miRNAs found in hESCs belong mostly to the miR-302 and miR-290 families expressed from miR-302/367 and miR-371–373 clusters, respectively [13,14]. Often referred to as *E*mbryonic *S*tem *C*ell *C*ycle (ESCC) miRNAs, they share common recognition “seed” sequences to target mRNAs. Functional studies of ESCC miRNAs have indicated that they are primarily required to allow the typical “uninterruptible” proliferation of stem cells by regulating G1 checkpoint control [14–17]. In contrast to the miRNAs required for differentiation, the survival of undifferentiated mouse ESCs is not affected by the absence of ESCC miRNAs [18]. Overexpression of miR-302 family members is able to reprogram human and mouse somatic cells to pluripotency [19,20].

In addition to miRNAs, miRNA-offset RNAs (moRNAs; moR’s; MORs) were recently reported in several next generation sequencing (NGS) data sets as a fraction of short RNA sequences mapping to *C*. *intestinalis*, mouse and human miRNA loci [21–27], reviewed in [28]. The function of moRNA sequences remains unknown and their location immediately adjacent to both miRNA 5p and 3p sequences has led to the suggestion that moRNAs may arise as by-products from Drosha/DGCR8-mediated cleavage of the pre-miRNA. Although moRNAs are expressed in relatively low levels compared to most miRNAs, they are developmentally expressed [22] and exhibit bias to arise upstream of miRNA loci [21–27] by either overlapping or starting sharply from the 5p miRNA’s 5’ end. Even though mature miRNAs are thought to be processed in cytoplasm, a large fraction of moRNAs were shown to locate in the nucleus [29] that may suggest either their transport from cytoplasm to nucleus similar to some nuclear enriched miRNAs [30,31] or nuclei-specific processing by nuclear small RNA synthesis enzymes [8,32–34].

Although the developmental expression, the association with conserved miRNAs and the preference for nuclear localization have been determined, the possible origin of moRNAs from cell type-specific miRNA loci, their molecular processing, mechanism of action and the biological function remain unknown. To deepen the understanding of moRNAs and their putative developmental stage-related expression, we utilized the highly cell type-specific miRNA profile of hESCs [13,14]. We prepared small RNA NGS libraries of two hESC lines and screened miRNAs and associated moRNAs in them and in two publicly available deep sequenced hESC small RNA libraries, and compared miRNA and moRNA expression profiles against NGS library constructed from human fibroblasts.

We report here that moRNAs are notably expressed in hESCs and show that hESC moRNAs with highest expression levels are represented by a common length isoform in distinct hESC lines. The most abundant moRNA expression was identified in the hESC libraries at the vicinity of hESC- and cancer-related miRNAs. In contrast to previous findings, the most abundant moRNA expression in hESCs was observed to predominate at the 3p region of the miRNA hairpin loci. Moreover, we report eight novel miRNAs representing the minor form of known miRNA precursors and seven novel miRNA hairpin structures.

## Results

### Small RNA sequence extraction workflow

**Fig 1:**
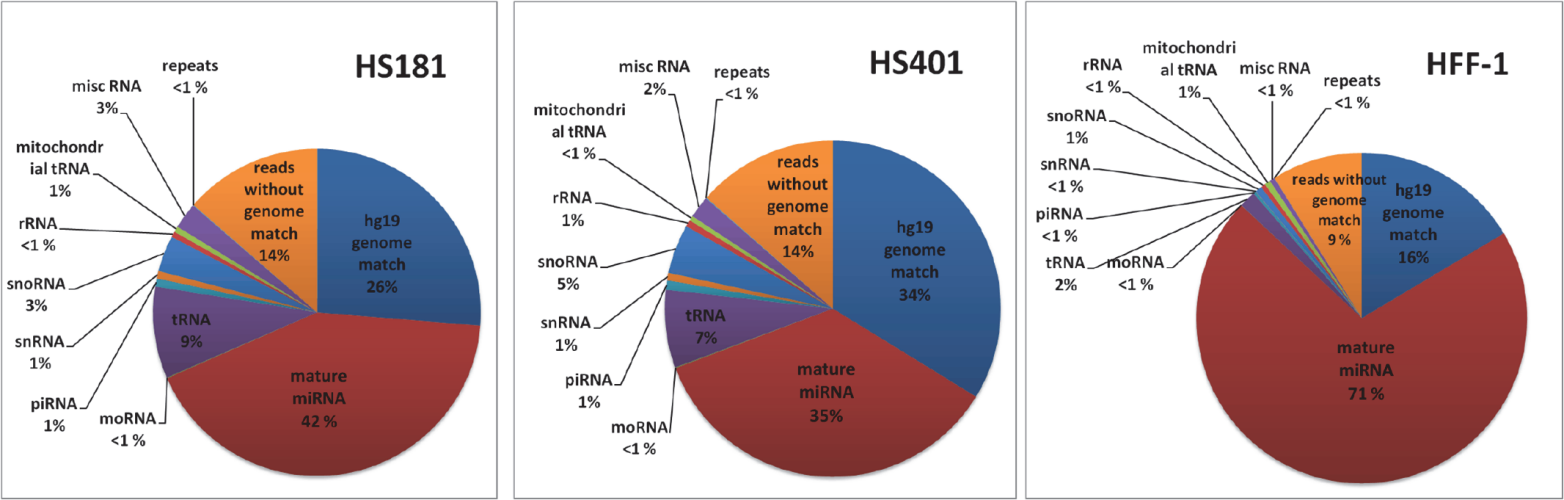
Distribution of reads according to RNA species annotations after preprocessing of raw reads in HS401, HS181, and HFF-1 lines. Pie charts illustrate under-representation of miRNA hairpin mapping reads in hESCs when compared to differentiated cells.

We deep-sequenced two short RNA libraries from hESC lines HS181 and HS401, and control HFF-1 library by using Illumina small RNA sequencing. The extracted reads were aligned to annotated small ncRNA loci and to hg19 human genome assembly as presented in Materials and Methods. The percentages of reads mapping to distinct classes of ncRNA in the three libraries are depicted in **Fig. 1** and the total numbers of mapped reads are provided in **S1 Table**. In each of the three samples, the majority of reads aligned to miRNA hairpin precursor sequences. This fraction dominated in HFF-1 in comparison with hESCs.

To extend small RNA characterization to additional hESC lines, we utilized data published by two small RNA deep sequencing projects: Morin et al. 2008 [35] (PRJNA79477) and Bar et al., 2008 [36] (GSE21722). In total, we analyzed four hESC libraries: HS401, HS181, H9 [35] and H1 [36], and three libraries of differentiated cells: HFF-1, embryoid bodies [35] and spontaneously differentiated hESCs [36]. To illustrate the read distribution through bioinformatics workflow in all libraries, the approximate read counts from the raw reads to miRNA sequence extraction are shown in **Table 1**. We observed relatively high abundance of miRNA-offset RNA (moRNA) loci mapping reads in the hESC libraries compared to cells with differentiated phenotype.

**Table 1:** Read counts and percentages compared to raw data in total of seven small RNA-seq libraries during bioinformatics workflow from raw reads to miRNA and moRNA sequences.

Reads	HS401	HS181	HFF-1	H9	EB	H1	Spont. diff.
Original lib	10.5M	11.1M	11.4M	6.1M	6.0M	0.28M	0.14M
Cleaned data	6.1M (59%)	8.4M (75%)	9.3M (82%)	4.8M (79%)	4.5M (75%)	0.22M (79%)	0.11M (79%)
Genome mapping	5.6M (53%)	7.5M (68%)	8.6M (75%)	4.5M (74%)	3.9M (65%)	0.20M (72%)	0.11M (76%)
miRNA hp mapping	2.2M (21%)	3.6M (32%)	6.6M (58%)	1.2M (19%)	1.2M (20%)	0.19M (68%)	0.10M (71%)
moR-5p mapping	3145 (0.030%)	3469 (0.031%)	983 (0.009%)	1412 (0.023%)	944 (0.016%)	171 (0.061%)	45 (0.032%)
moR-3p mapping	1961 (0.019%)	2806 (0.025%)	41 (<0.001%)	982 (0.016%)	855 (0.014%)	34 (0.015%)	15 (0.011%)

Reads were aligned to reference sequences by allowing two mismatches.

### Expression of known miRNAs

To characterize dominating miRNAs in hESCs, miRNA expression was profiled from the three libraries prepared by us: HS401, HS181 and HFF-1. miRNA differential expression analysis between hESC and HFF-1 libraries resulted in 344 miRNAs significantly differentially expressed **(S2 Table)**. Of these, 271 miRNAs were overexpressed in hESCs and 73 were overexpressed in HFF-1. The fifteen most significant miRNAs derived from eleven different hairpin precursors, all overexpressed in the hESC libraries, are shown in **Table 2**. Ten of these miRNAs belong to known hESC-specific miR-302/367 and miR-371/372/373 clusters. Known hESC miRNA cluster miR-106a-363, its paralog miR-17-92 and a large C19MC cluster located at close vicinity of miR-371/372/373 were also found to produce significantly overexpressed miRNAs [4–6,13–17,35] **(S2 Table)**. Differential expression of three overexpressed and two underexpressed miRNAs were verified by quantitative Real-Time PCR (qRT-PCR) **(S1 Fig.)**.

**Table 2:** Lists of 15 most overexpressed miRNAs in hESC lines when compared to HFF-1 foreskin fibroblast by statistical computing using DEseq algorithm.

miRBase name v18	HS401 reads (rpm)	HS181 reads (rpm)	HFF-1 reads (rpm)	P value	Location
hsa-miR-302a-5p	16380,51	23920,20	18,15	8,39E-31	miR-302 cluster
hsa-miR-363-3p	8482,25	6651,71	4,71	2,89E-30	miR-106a-363
hsa-miR-302d-3p	5134,52	5504,53	3,30	1,98E-29	miR-302 cluster
hsa-miR-302a-3p	7597,99	6680,41	5,18	1,63E-29	miR-302 cluster
hsa-miR-302b-3p	6556,53	6090,26	4,83	5,23E-29	miR-302 cluster
hsa-miR-302c-3p	5399,63	4615,92	4,12	5,29E-28	miR-302 cluster
hsa-miR-372	5522,03	1845,79	2,36	1,40E-27	miR-371/372/373 cluster
hsa-miR-371a-5p	6083,84	3410,09	4,95	9,90E-27	miR-371/372/373 cluster
hsa-miR-200c-3p	2240,66	2437,46	2,47	9,51E-25	miR-200c/miR141
hsa-miR-367-3p	1111,78	997,75	0,35	3,35E-24	miR-302 cluster
hsa-miR-20b-5p	598,48	504,74	0,24	1,63E-20	miR-106a-363
hsa-miR-302b-5p	539,25	809,27	0,71	4,26E-20	miR-302 cluster
hsa-miR-20b-3p	508,98	506,95	0,47	5,32E-19	miR-106a-363
hsa-miR-106a-5p	434,71	389,66	0,24	8,18E-19	miR-106a-363
hsa-miR-302d-5p	275,26	331,99	0,12	5,27E-18	miR-302 cluster

Normalized read count (RPM), P-value and the name of surrounding miRNA cluster (location) are shown for each miRNA. Relative expression analysis was made using reads mapping to the genome allowing 2 mismatches.

In addition, we found eight novel 5p or 3p miRNAs from known hairpin precursors which previously contained the reference sequence for only one mature miRNA in miRBase **(Table 3)**. Three of the novel miRNAs were detected in both hESCs and HFF-1, while five were expressed specifically in hESCs. The average normalized read count of the most abundant isomiR of the hESC-specific novel miRNAs ranged from 2.1 to 136.9 reads per million mapped reads (RPM), and their expression appeared to be consistent in the hESC lines. Moreover, from the set of reads not annotated to any RNA species, we found seven novel miRNA hairpins, two of which are conserved in mammals **(Table 4)**. For all of the novel hairpins, mature miRNA expression was observed only from either 5p or 3p stem, and three of them could not be detected in fibroblasts.

**Table 3:** New mature miRNA sequences found from known miRNA hairpin precursors.

Novel miRNA	Sequence	HS401	HS181	HFF-1
hsa-miR-135a-2-3p	TGTAGGGATGGAAGCCATGAA	152.30	121.56	0
hsa-miR-137-5p	ACGGGTATTCTTGGGTGGATAA	0.4	0.4	3.4
hsa-miR-1912-5p	CTCATTGCATGGGCTGTGTATA	1.9	1.2	0
hsa-miR-498-3p	AAAGCACCTCCAGAGCTTGAAGC	2.6	1.8	0
hsa-miR-519a-2-5p/has-miR-520b-5p	CCTCTACAGGGAAGCGCTTTC	1.5	1.8	0.1
hsa-miR-520e-5p	CTCAAGATGGAAGCAGTTTCTG	3.0	1.1	0
hsa-miR-526a-1-3p	GAAAGCGCTTCCTTTTAGAGGA	5.8	3.2	0
hsa-miR-549a-5p	AGCTCATCCATAGTTGTCACTG	0.2	0.6	6.1

The sequence and the normalized read count (RPM) of most abundant isomiR are shown in the table for each library.

**Table 4:** Novel miRNA hairpins found, their dot-bracket—notation, genomic loci and conservation.

hairpin	chr	strand	start	end	hairpin sequence		HS401		HS181		HFF1	Cons.
name						5p	3p	5p	3p	5p	3p	
hsa-mir-10522	5	-	17156011	17156121	AGAAGAATTGGCCTACTCAGGtgtcagcaaagccttcctctcTGAATAGGTCAATTCCCCTCA	14	0	9	0	0	0	no
					....(((((((((((.((((.....................)))).)))))))))))......							
hsa-mir-10523	5	+	172342066	172342178	GACAATGATGAGAAGACCTGAGGAtttgcagcccccagccctgggttcaagtcCCAGCTCTACCCCTTCTTGGCCC	25	0	50	0	8	0	no
					..(((.((.....(((.(((.(((((((.(((((........))))))))))))))).)))......)))))....							
hsa-mir-9983	6	+	39042066	39042177	GGAAATGTTCTAGCCAAAAAAGtttgccaagaaccattgtgtctttTTTTTTGCTGGAACATTTCTGG	0	12	0	17	0	0	yes
					((((((((((((((.(((((((...(((((......))).))...)))))))))))))))))))))..							
hsa-mir-10524	6	-	79248939	79249046	cCAGGATGCCAGCATAGTgagttcTGGTGAGGGCTGTTTTCCTGGTT	48	0	167	0	0	0	no
					((((((.(((..(((((......)).)))..))).....))))))..							
hsa-mir-10525	7	+	74010263	74010373	AAGGTGTATGATGGGACTtgggagaaagtactcccgggTGACTATGATGTGCACCTGAT	0	11	0	4	0	3	no
					.((((((((.((((.(((((((((......)))))))))..))))...))))))))...							
hsa-mir-10526	11	-	122022791	122022900	TCCCCTTAGTTTCCTTTAAGagtgataaaaatggAAAAGGGGGCTGAGGTGGAG	0	2	0	2	0	23	no
					(((((((((((((((((....((.......))...)))))))))))))).))).							
hsa-mir-10527	12	+	64217423	64217534	AAAGCAAATGTTGGGTGAACGGCtgtttcctcttattcaagcCATGCACCTTACTCTTGCTGGTA	8	0	24	0	83	0	yes
					..(((((..((.(((((...((((...............))))...))))).))..)))))....							

Mature miRNAs are indicated by caps and underlined, and the raw number of 5p and 3p reads found in each library are shown.

### miRNA-offset RNAs expression profile in hESCs

**Fig 2:**
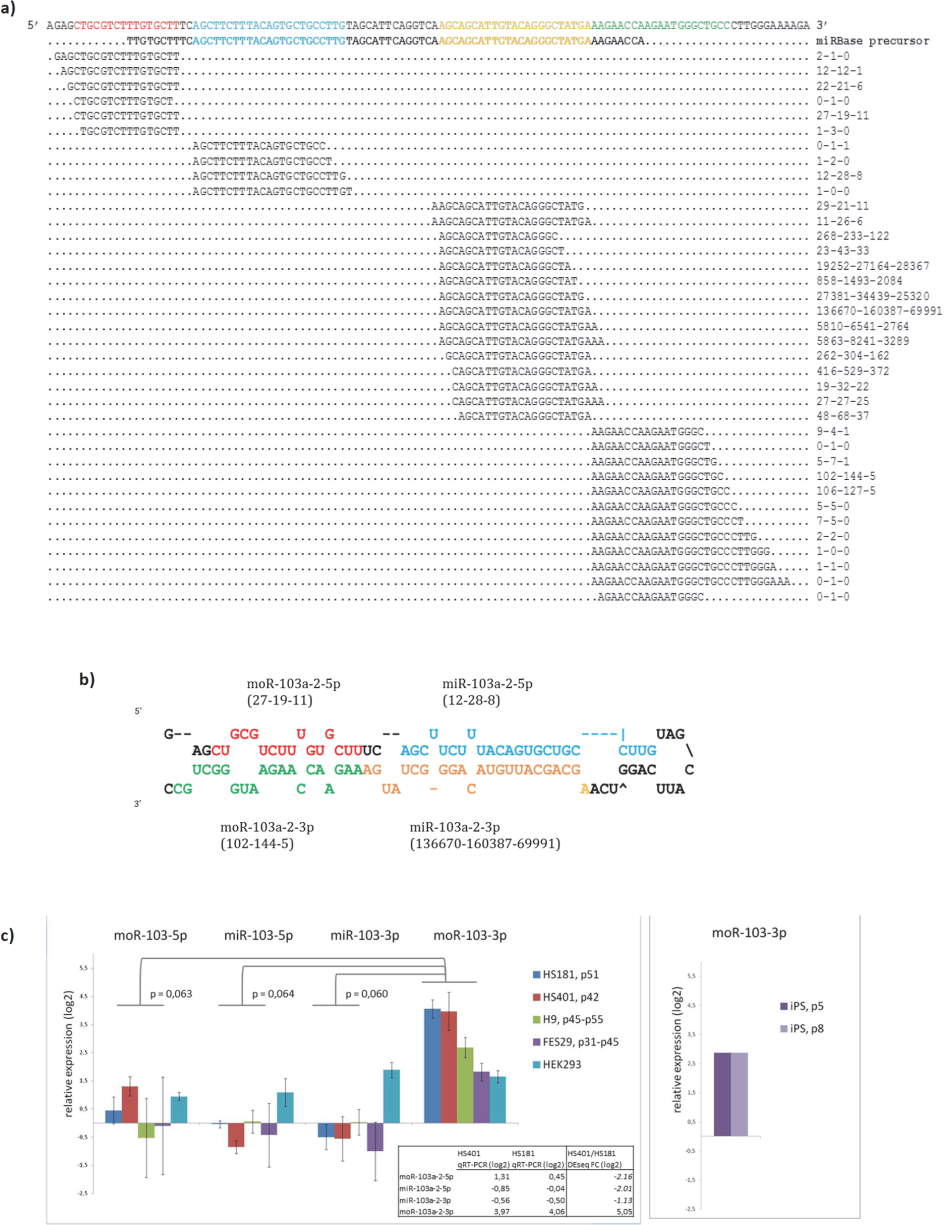
Analysis of pre-miR-103a-2 small RNA derivatives. a) Expression of unique isomiR and isomoR reads from the extended precursor of hsa-mir-103a-2. The first line indicates the predicted precursor based on moRNA reads. The second line **bolded** shows the miRBase hairpin precursor. The colors indicate mature products: red = moR-5p, blue = miR-5p, orange = miR-3p, green = moR-3p. Values on the right side indicate the raw counts of each read found from different libraries, order is HS401—HS181—HFF-1. All of the observed moR-103a-2 isomoRs are shown, but only the most abundant isomiR reads are shown. b) The minimal free energy structure of the extended hairpin sequence of hsa-mir-103a-2 (mfe = -37.50 kcal/mol). c) qRT-PCR of pre-miR-103a-2 hairpin derived small RNAs. Bars indicate logarithmic fold change relative to HFF-1 fibroblasts with Standard Deviation (SD; number of replicates n = 3 for HS401, HS181, FES29, HEK293; n = 2 for iPSC p5, iPSC p8).

**Fig 3:**
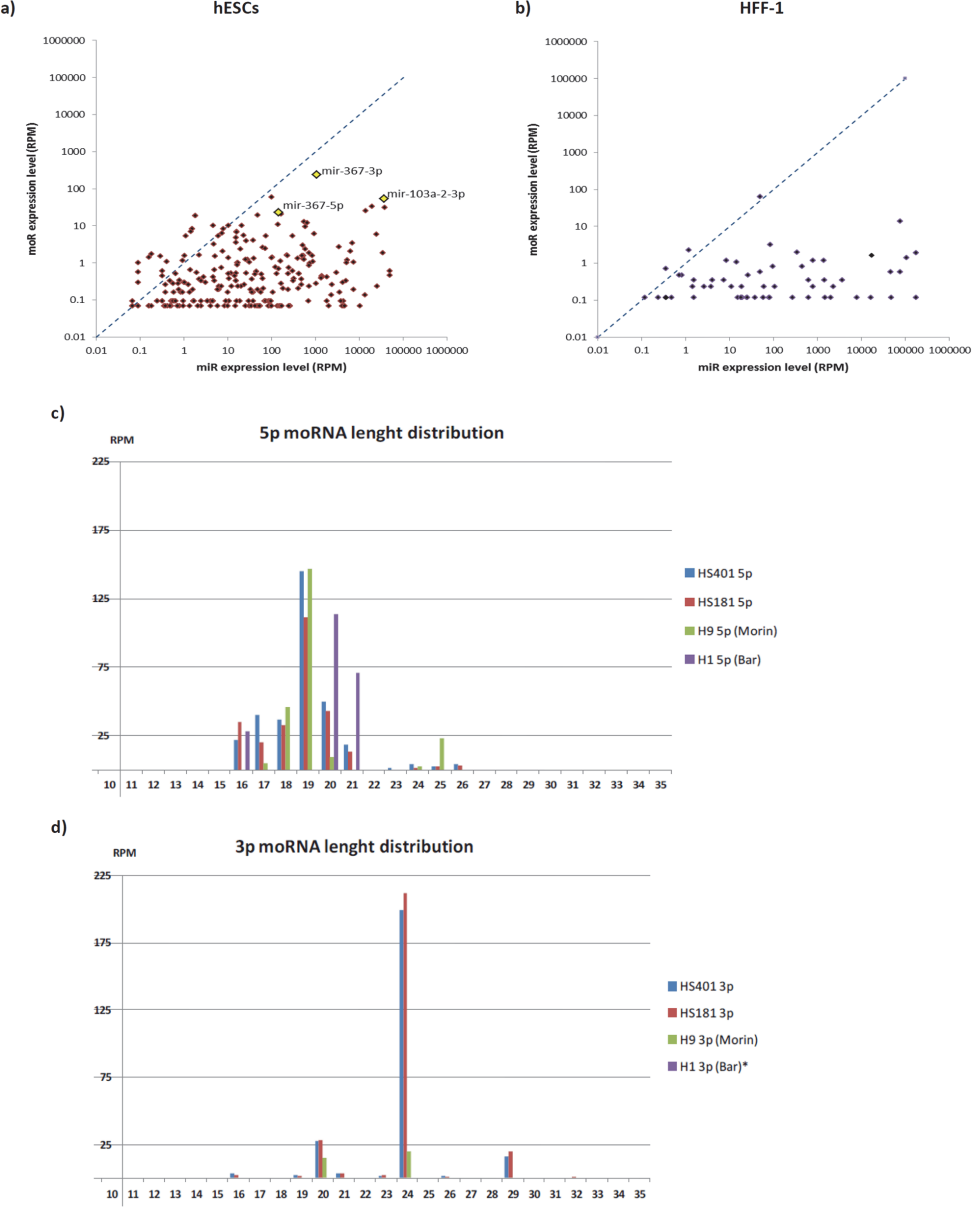
Analysis of miRNA and moRNA correlation, and moRNA lenght distribution. Scatter plots of expression levels (reads per million; RPM) of miRNAs vs. moRNAs derived from common extended hairpin precursor arms are shown in a) hESCs and b) HFF-1. Spearman correlation coefficient for hESC data is 0.219 (p-value = 0.000231) and for HFF-1 0.381 (p-value = 4.43e-11). Only reads where the RPM value of both miR and moR is greater than 0.5 are shown. c) moR-5p and, d) moR-3p length distributions from hESC lines HS401, HS181, H9 (Morin et al., 2008) and H1 (Bar et al., 2008) are shown in bar graphs. *moR-3p reads were not detected in H1 data.

The identification of abundant offset sequences mapping at the hESC miRNA loci prompted us to perform differential expression analysis following the calculation of candidate moRNAs. In total, 350 moRNAs were identified in our data: 220 5’ moRNAs (moR-5p) and 130 3’ moRNAs (moR-3p) expressed with at least one read from the vicinity of 273 miRNA hairpins **(Table 5, S3 Table)**. 326 moRNAs were found in hESCs, while only 65 were found in HFF-1 cells. The length of detected moRNA reads was between 15–36 nt (median = 20 nt), and similar to miRNAs, moRNAs were expressed with overlapping, variable length reads, called isomoRs **(Fig. 2 A,B)**. We measured the expression of each moRNA by counting the number of reads that aligned to the locus of its most abundant isomoR sequence allowing at most two mismatches and extension by two nucleotides both upstream and downstream. Of all the reads deriving from miRNA haipin loci, the proportion of moRNA reads was roughly five times larger in hESCs than in HFF-1 whereas the percentage of mature miRNA reads in hESCs was only about half of their fraction in HFF-1 **(Table 1, S1 Table)**. Also the expression level of distinct moRNAs was higher in hESCs (median 0.5, average 3.7 RPM) than in HFF-1 (median 0.2, average 1.5 RPM). In hESCs, 58% and in HFF-1, 96% of the moRNA reads derived from the 5’ arm of the hairpin which suggests a bias towards 3’ moRNA expression in hESCs. Considering the total of 350 moRNAs found, 229 (65%) were derived from a conserved miRNA hairpin loci (conserved in mammals, mean PhastCons value > 0.5). The extended hairpins (moR-5p+hairpin+moR-3p) were also conserved in 223 cases (64%). Consistent with earlier studies, we did not detect any clear correlation between the expression levels of mature miRNAs and moRNAs derived from the same miRNA hairpin arm in neither hESCs nor HFF-1 **(Fig. 3 A,B)**. In addition, seven of the 12 most significant 5’ moRNAs shown in **Table 5** are expressed from a miRNA hairpin opposite to the major miRNA stem (3p) and one of them, moR-421-5p, is expressed alone without expression of its adjacent miRNA miR-421. This phenomenon where the 5’ moRNA is expressed from the same arm with the minor miRNA has been recently observed also by Gaffo and co-workers [37]. On the other hand, all the three most significant 3’ moRNAs derive from precursors where also the major miRNA is expressed from the 3p arm.

**Table 5:** moRNA frequencies (moR count / millions of exactly mapped reads) in four different human hESC libraries, two day 15 libraries (EB and adherent, spontaneously differentiated) and HFF-1.

moR name	most abundant isomoR sequence	HS401	HS141	H9	H1	HFF-1	EB	spont.diff
hsa-moR-367-3p	TGGATTGTTAAGCCAATGACAGAA	196.6	209.3	19.4	-	0.1	2.4	-
hsa-moR-103a-2-3p	AAGAACCAAGAATGGGCTGC	24.5	23.5	14.6	-	0.7	17.5	-
hsa-moR-92a-1-3p	TGAGTTTGGTGGGGATTGTGACCAGAAGA	15.8	19.9	-	-	0.1	-	-
hsa-moR-16-2-3p	GTGTGACAGGGATACAGCAAC	3.6	3.4	1.3	-	-	-	-
hsa-moR-21-3p	TGACATTTTGGTATCTTTCA	3.1	2	0.4	-	-	-	-
hsa-moR-221-3p	AGGCTACCTGGAAACATGTTCTCC	2.4	2.4	-	-	-	-	-
hsa-moR-103a-1-3p	AGGCATTGAGACCTGTTCT	2.2	1.6	1.3	-	0.1	-	-
hsa-moR-20b-3p	ACTCTTGGATAACAAA	1.7	2.1	0.9	-	-	-	-
hsa-moR-421-5p	ATCCGGTGCACATTGTAGGC	26.9	23	2.2	-	0.3	-	-
hsa-moR-21-5p	TGTACCACCTTGTCGGG	24.5	12.2	3.9	-	9.7	2.4	137.9
hsa-moR-302a-5p	CAAGACTGGGCTCCCCACC	14.9	15	31.4	70.9	-	-	-
hsa-moR-363-5p	ATGATCTGTTTTGCTGTTG	14.2	11.7	4.7	4.7	0.1	6.3	-
hsa-moR-367-5p	CTTGGCTACAGGCCAT	13.9	31.8	2.6	-	-	3.9	-
hsa-moR-517a-5p	GAAGATCTCAGGCAGTGAC	12.5	9.8	2.2	-	-	1.9	-
hsa-moR-517c-5p	GAAGATCTCAGGCAGTGAC	12.5	9.8	-	-	-	1.9	-
hsa-moR-296-5p	AGGAGAAAGGACCCTTCCA	10.3	10.8	-	-	0.3	-	-
hsa-moR-17-5p	TGACCAGTCAGAATAATG	9.6	3.8	-	-	-	1	-
hsa-moR-20a-5p	ATGTGACAGCTTCTGTAGCAC	9.4	8	0.9	-	-	1.9	-
hsa-moR-33a-5p	CCTGGCGGGCAGCTGTG	9.4	5.4	14.2	-	2.5	5.4	9.2
hsa-moR-515-1-5p	AGAAGATCTCATGCAGTCA	9.4	2.6	0.4	-	-	-	-
hsa-moR-515-2-5p	AGAAGATCTCATGCAGTCA	9.4	2.6	0.4	-	-	-	-
hsa-moR-519e-5p	AGAAGATCTCATGCAGTCA	9.4	2.6	0.4	-	-	-	-
hsa-moR-1283-2-5p	AGAAGATCTCAAGCTGTGA	6.7	6.7	32.3	-	-	9.7	-
hsa-moR-525-5p	AGAAGATCTCAAGCTGTGA	6.7	6.7	32.3	-	-	9.7	-
hsa-moR-518a-1-5p	AGAAGATCTCAAGCTGTGA	6.7	6.7	32.3	-	-	9.7	-
hsa-moR-520d-5p	AGAAGATCTCAAGCTGTGA	6.7	6.7	32.3	-	-	9.7	-
hsa-moR-527-5p	AGAAGATCTCAAGCTGTGA	6.7	6.7	32.3	-	-	9.7	-
hsa-let-7f-2-5p	ACACTGGTGCTCTGTGGGA	6.7	5.5	11.2	9.5	0.4	5.4	9.2
hsa-moR-625-5p	TGGTAAGGGTAGAGGGATG	6.7	4.7	-	-	0.1	-	-
hsa-moR-103a-2-5p	CTGCGTCTTTGTGCTT	6.5	3.1	4.3	9.5	1.6	1.5	46
hsa-moR-125a-5p	ATGTTGCCAGTCTCTAGG	5.8	3.8	0.4	-	1.3	-	-
hsa-moR-302b-5p	TGTTGGGTGGGCTCCCTTCA	5.3	4.2	8.6	113.5	-	2.4	-
hsa-moR-4521-5p	GTGGGTTCGAATCCCATCCTCGTCGG	4.3	3.1	23.2	4.7	1.3	17.5	-
hsa-moR-221-5p	TGAACATCCAGGTCTGGGGCATGA	4.3	1.3	-	-	0.3	-	-
hsa-moR-516a-2-5p	AGAAGATCTCAGGTTGTGACC	3.8	2.1	0.4	-	-	1	-
hsa-moR-519a-1-5p	AGAAGACCTCAGGCTGTGAC	3.4	1	1.3	-	-	-	-
hsa-moR-324-5p	CTGAGCTGACTATGCCTCCC	3.4	0.8	-	-	0.1	0.5	-
hsa-moR-197-5p	GGAATCTGTGCTCTGGGGGCTGTGC	2.6	2.3	2.6	-	0.4	2.4	9.2
hsa-moR-524-5p	AGAAGATCTCATGCTGTGACC	2.6	1.5	1.3	-	-	-	-
hsa-moR-518f-5p	AGAAGATCTCATGCTGTGACC	2.6	1.5	1.3	-	-	-	-
hsa-moR-302d-5p	ATCTGTTAAGGGGCCCCCTC	2.4	1.8	3	4.7	-	-	-
hsa-moR-128-1-5p	GTTCCTGAGCTGTTGGAT	2.2	3.4	-	-	0.1	0.5	-
hsa-moR-876-5p	ACAAACTGTGAAGTGCTGTG	2.2	3.1	1.7	-	-	-	-
hsa-moR-519a-2-5p	AGAAGATCTCAGGCTGTG	1.9	3.4	4.3	-	-	1	-
hsa-moR-522-5p	AGAAGATCTCAGGCTGTG	1.9	3.4	4.3	-	-	1	-
hsa-mor-520a-5p	AGAAGATCTCAGGCTGTG	1.9	3.4	4.3	-	-	1	-
hsa-moR-521-1-5p	AGAAGATCTCAGGCTGTG	1.9	3.4	4.3	-	-	1	-
hsa-moR-18a-5p	ATGTTGAGTGCTTTTTGT	1.9	2.8	0.9	-	-	0.5	-
hsa-moR-106b-5p	CGCTCCAGCCCTGCCGGGGC	1.9	1.6	-	-	-	-	-
hsa-moR-370-5p	GGGGCACAAGACAGAGAAGC	1.9	1.5	0.4	-	4.5	0.5	-
hsa-moR-191-5p	GCCAACGGCTGGACAGCGGG	0.7	1.6	-	-	0.3	-	-
hsa-moR-92a-2-5p	ACTCATGCCCATTCATCCC	0.7	1.6	-	-	-	0.5	-
hsa-moR-214-5p	AGAGTTGTCATGTGTC	0.2	0.7	-	-	1.5	-	-
hsa-moR-27a-5p	CTGTGCCTGGCCTGAGGAGC	0.2	0.3	0.4	-	1.5	-	-

Shown are moRNAs supported with min 10 reads in at least one of the libraries, no mismatches were allowed.

Also in line with previous studies, we observed that 5p moRNA reads exhibit a length distribution at 16–26 nt with a peak at 19 nt, whereas 3p moRNA reads were distributed more randomly between 16 and 29 nt **(Fig. 3 C,D)**. However, the scattered distribution of 3’ moRNAs can partly be explained by the low number of unique moR-3p sequences. There were 26 moRNAs, derived from 24 miRNA hairpin loci, whose most abundant isomoR was detected with more than 5 RPM and 11 of them were expressed with more than 10 RPM **(Table 5, S3 Table)**. On the other hand, up to 159 (45%) of the moRNA sequences were found only once in our data. The identified moRNAs were further searched from H9 data by Morin et al. 2008 [35] and from H1 data by Bar et al. 2008 [36] **(S3 Table)** from which we found 106 and 21 moRNAs in common with our data, respectively. While Morin et al. data contained moRNAs from both arms of 11 miRNA hairpins, we detected only 5’ sequences from Bar et al. data. Of the most abundant isomoRs, 32 in Morin et al. data and four in the Bar et al. data were exactly the same as our reference sequence for the particular moRNA.

We found 57 moRNAs to be significantly differentially expressed between the hESC and HFF-1 libraries **(Table 6, S4 Table)**, and all of them were overexpressed in hESCs. Among them are seven of the ten moRNAs that derive from the pluripotency related miR-302/367 cluster. The other known hESC miRNA clusters detected highly represented in our study gave rise to several overexpressed moRNAs as well: moRNAs derived from both ends of mir-363 and miR-20b hairpins, moR-92a-2-5p from miR-106a-363 cluster and four moRNAs from the paralog miR-17-92 cluster. Also C19MC cluster was represented by 20 moRNAs, which all derived from miR-515 family of hairpins. Moreover, many of the differentially expressed moRNAs found in our data sets were also present in H9 and H1 data. In conclusion, moRNAs appeared to arise from highly expressed and hESC-selective miRNA clusters with a couple of exceptions such as miR-371/372/373 cluster with high miRNA expression in hESCs but only few detected moRNAs. On the other hand, only few reads of moR-103a-2-3p **(Fig. 2)** in HFF-1 made it overexpressed in hESCs, while its related miRNA miR-103a-3p, was highly expressed in all libraries.

**Table 6:** List of 15 most overexpressed moRNAs in hESC lines when compared to HFF-1 foreskin fibroblast by statistical computing using DEseq algorithm.

name	HS401 reads (rpm)	HS181 reads (rpm)	HFF-1 reads (rpm)	P value	Location
hsa-moR-367-3p	226,38	250,99	0,12	1,71E-95	miR-302 cluster
hsa-moR-302a-5p	32,34	34,08	0,12	9,46E-20	miR-302 cluster
hsa-moR-103a-2-3p	57,35	51,61	1,65	1,45E-18	miR-103-2a/b cluster
hsa-moR-367-5p	15,04	31,18	0	6,63E-18	miR-302 cluster
hsa-moR-92a-1-3p	24,44	26,77	0,12	1,50E-17	miR-17-92 cluster
hsa-moR-421-5p	29,52	27,60	0,35	1,67E-16	miR-374b/421 cluster
hsa-moR-517a-5p	21,25	15,59	0	2,76E-15	C19MC
hsa-moR-517c-5p	21,25	15,59	0	2,76E-15	C19MC
hsa-moR-363-5p	21,43	20,70	0,12	2,92E-15	miR-106a-363 cluster
hsa-moR-296-5p	19,37	18,77	0,24	9,68E-14	miR-296/298 cluster
hsa-moR-302b-5p	13,54	10,35	0	2,18E-12	miR-302 cluster
hsa-moR-1283-2-5p	9,78	10,35	0	1,50E-11	C19MC
hsa-moR-525-5p	9,78	10,35	0	1,50E-11	C19MC
hsa-moR-20a-5p	10,34	8,83	0	2,43E-11	miR-17-92 cluster
hsa-moR-518a-1-5p	6,96	9,52	0	5,87E-11	C19MC

Normalized read count (RPM), P-value and the name of surrounding miRNA cluster (location) are shown for each moRNA. Relative expression analysis was made using reads mapping to the genome allowing 2 mismatches.

### Probing for the functionality of moRNAs

On average, 97% of the isomoR reads related to each 3’-moRNA had a consistent 5’ end, which may refer to importance of the 5’ part of the sequence in target recognition (for miRNAs, this fraction was ∼80% for both stems in our data). This observation led us to study the possibility of ‘miRNA-like’ function for moRNAs using moR-103a-2-3p which is one of the most abundantly expressed moRNAs in hESCs and derives from the locus of mir-103a-2, a conserved regulator of cancer metastasis, cell proliferation, insulin response control and adipogenesis [38–41]. Hence the function of mir-103a is well-known and, in addition, its hairpin structure was found to be associated with moRNAs in HS401, HS181 and H9 (Morin et al. data) and also in previously published human moRNA reports [23,26].

First, we ensured the hESC-related expression of moR-103a-2-3p using quantitative Real-Time PCR (qRT-PCR). The expression of all four small RNA derivatives from pre-miR-103a-2 hairpin precursor was measured in four different hESC lines including two independent hESC lines H9 and FES29, and in two human induced pluripotent stem cell (hiPSC) lines HEL23 and HEL41 in early passages. In addition, HEK293 cell line was analyzed as a reference **(Fig. 2 C)**. Overexpression of moR-103a-2-3p, but not overexpression of any other derivatives, was detected in all analyzed lines when compared with HFF-1. The results are in line with the moRNA differential expression analysis made from NGS data **(Table 6)**.

Next, we studied the effect of transfection of HFF-1 cells with moR-103a-2-3p using transfection with miR-103a-3p as a positive control. The whole transcriptome microarray analysis for the transfected cells indicated that the moR-103a-2-3p mimic regulated either directly or indirectly the expression of over 650 genes at least 2 fold (117 up and 538 down, p: ≤0.05; **S5 Table**). The transfection with the positive control, miR-103a-3p, caused regulation of about 700 genes at least 2-fold (216 up and 497 down, p: ≤0.05). A notable fraction (321 of 538/497) of the down-regulated genes was common to both moR-103a-2-3p and miR-103a-3p transfections. Thus moRNA transfection affected the mRNA expression levels in HFF-1 cells, and the effect seemed to be partly similar with the effect of miRNA transfection. To elucidate if moRNA has miRNA supporting function via modulation of its expression, we ran qPCR to quantitate mature miR-103a-3p after transfection of moR-103a-2-3p to HFF-1. However, the miR-103a-3p expression level remained unchanged (data not shown), indicating that moRNA-mediated modulation of miRNA expression does not explain the common down-regulated genes.

The large amount of genes down-regulated by both mimics could also be because moR-103a-2-3p acts directly as a post-transcriptional regulator and shares at least part of its targets with miR-103a-3p. Because moR-103a-2-3p sequence did not have any perfect matches to other places in the genome, and because 98% of its isomoRNAs shared the same 5’-part, we deduced that moR-103a-2-3p might work similarly with miRNAs and down-regulate genes with minimum of 7-mer seed match in their 3’ UTRs. To test this possibility, we considered a gene with a perfect 7-mer seed (nucleotides 2–8 from the 5’ end) match of miR-103-3p or moR-103a-2-3p in its 3’ UTR as a putative target of that small RNA and predicted the targets for these two in the three distinct gene sets: genes down-regulated by both mimics, genes down-regulated by only miR-103a-3p and genes down-regulated by moR-103a-2-3p **(Table 7, S7 Table)**. To be conservative, we conducted the target analysis only for those genes that had an annotated 3’UTR in GRCh37/hg19. The number of putative miR-103a-3p targets was largest in the gene group that was down-regulated by miR-103a-3p but not by moR-103a-2-3p (50 genes; 30% of all genes in this group), while in that group the number of moR-103a-2-3p ‘seed’ matches was smallest (9; 5%). On the other hand, the number of genes with moR-103a-2-3p ‘seed’ matches was largest (43; 27%) in the group of genes which was down-regulated only by moR-103a-2-3p but not by miR-103a-2-3p, while the number of putative miR targets in that group was smaller (30; 19%) than the number of moR ‘targets’. Hence, there seemed to be a connection between the existence of a moR ‘seed’ match in the gene 3’ UTR and its down-regulation by moRNA. In all groups, the number of genes that had matches for both miR and moR was rather low, being about 4%.

**Table 7:** Number of predicted miR-103a-3p and moR-103a-2-3p targets in the sets of genes down-regulated by both mimics, down-regulated by only miR-103a-3p and down-regulated by moR-103a-2-3p.

Gene set	Down by both mimics	Down by miR only	Down by moR only
genes with annotated 3’UTR	226	165	158
miR seed match	**46**	**50**	30
moR ‘seed’ match	29	9	**43**
seed matches for both	9	7	7

The analysis was made only for genes with an annotated 3’ UTR in GRCh37/hg19.

**Fig 4:**
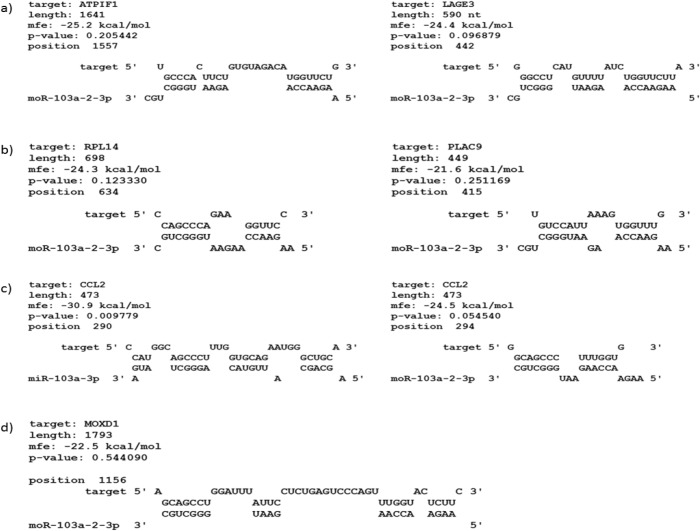
Examples of predicted binding sites for moR-103a-2-3p. a) perfect moR-103a-2-3p ‘seed’ matching sites, b) predicted minimum free energy sites for moR-103a-2-3p, c) common predicted site for miR-103a-3p and moR-103a-2-3p, d) predicted minimum free energy site for moR-103a-2-3p.

Next, we inspected the number of possible miR-103a-3p and moR-103a-2-3p binding sites in the 3’ UTRs of the most significantly down-regulated genes in both of the transfection studies **(Table 8** and **Table 9)**. Eight of the ten genes most significantly down-regulated by miR-103a-3p had at least one seed match for miR-103a-3p in their 3’ UTRs while one of them, C7orf55, contained a 7-mer seed match also for moR-103a-2-3p **(Table 8)**. Because C7orf55 was down-regulated by the moR mimic where its fold change (FC = -3.5) was about the same as with miR-103a-3p (FC = -3.6), it could also be a target of moR-103a-2-3p. While the existence of miRNA seed matches in this gene group was expected, the absence of moR seed matches was also noteworthy. Of the top ten genes down-regulated by moR-103a-2-3p, five contained one or two moR seed matches in their 3’ UTRs but only four of them did not contain any miR seed matches **(Table 9, Fig. 4 A)**. Because five of the genes significantly down-regulated by moR-103a-2-3p did not contain any perfect seed matches for it, we predicted its minimum free energy (mfe) binding sites in their 3’UTRs with RNAhybrid **(Fig. 4)** [42]. Typically, an mfe moR-103a-2-3p binding site contained a 5–6nt 5’ seed match and a continuous 7 nt stretch binding to the moR 3’ end **(Fig. 4 B-D)**. We observed also an example of predicted mfe site common for both miR-103a-3p and moR-103a-2-3p **(Fig. 4 C)**.

**Table 8:** The ten most significantly down-regulated genes in miR-103a-3p transfection study.

Rank miR	Gene name	FC miR	FC moR	miR seeds	moR seeds
1	TRIAP1	-4.8	ns	1	0
2	LY6E	-4.6	-2.2	2	0
3	ARL2	-4.1	ns	3	0
4	HCFC1R1	-4	-2.1	0	0
5	BCAT2	-3.7	ns	1	0
6	SMPD1	-3.6	ns	3	0
7	C7orf55	-3.6	-3.5	2	1
8	HIST1H2BD	-3.5	ns	0	0
9	TMEM256	-3.5	-4.3	1	0
10	ZNF428	-3.4	-2.1	1	0

Fold changes of the genes in both transfection studies, and the number of perfect 7mer seed matches found in their 3’ UTRs for both miR and moR are shown. (ns = expression of the gene is not significantly changed).

**Table 9:** The ten most significantly down-regulated genes in moR-103a-2-3p transfection study.

Rank moR	Gene name	FC miR	FC moR	miR seeds	moR seeds
1	ATPIF1	-2.1	-7.8	0	1
2	RPL14	-2.7	-6.4	2	0
3	LAGE3	-3.2	-6.2	0	1
4	PLAC9	-3.2	-5.7	1	0
5	CCL2	ns	-5.4	0	0
6	ZMAT2	-2.1	-5.3	1	2
7	ADIRF	-2.8	-5.1	0	0
8	POLR2G	-2.2	-5.1	2	2
9	HSPE1	-2.1	-5.0	1	1
10	MOXD1	ns	-4.8	1	0

Fold changes of the genes in both transfection studies, and the number of perfect 7mer seed matches found in their 3’ UTRs for both miR and moR are shown. (ns = expression of the gene is not significantly changed).

Further, in order to gain global view of the possible function of moR-103a-2-3p, we performed Gene Ontology (GO) analysis with DAVID Functional Annotation Clustering-tool [43] for genes that were down-regulated only with moR-103a-2-3p mimic, only with miR-103a-3p mimic, or with both mimics **(S6 Table)**. The ten most significantly enriched GO terms in the gene set down-regulated only by moR-103a-2-3p were related to ribosome, translation or mitochondria, and were the same as the top ten GO terms enriched among the genes that were down-regulated by both mimics **(Table 10)**. In the strongly enriched category “ribosome”, most of the genes encoded either ribosomal pseudogenes or mitochondrial ribosomes (36 of 41 genes in gene set down-regulated by both mimics). The miR-103a-3p down-regulated genes were also enriched in nine top categories shown in **Table 10**. In addition, enriched only in this gene set were terms “nucleosome” (p = 2.5E-4), “protein-DNA complex” (p = 1.1E-3), “chromatin assembly” (p = 7.7E-4), “phosphatidylethanolamine (lipid) binding” (p < 3.8E-4) and “fatty acid catabolic process” (p = 0.036). The lipid and fatty acid-associated categories reflect to the known functions of miR-103a-3p, while the chromatin-associated down-regulated categories may indicate still unknown functions for it. In all, the microarray analysis showed that miR-103a-3p has functions that are not connected to the function of moR-103a-2-3p. On the other hand, it did not show any specific functions for moR-103a-2-3p, but instead suggested that it might function in concert with miR-103a-3p.

**Table 10:** p-values for the top 10 enriched GO terms for genes that were down-regulated with both mimics.

GO term	Down by both mimics p-value	Down by moR only p-value	Down by miR only p-value
structural constituent of ribosome	3.9E-34	5.5E-10	6.0E-5
ribosome	4.9E-33	3.1E-8	7.1E-4
ribosomal subunit	2.9E-32	5.9E-8	1.5E-4
ribonucleoprotein complex	1.7E-30	4.0E-11	2.3E-3
translation	1.8E-25	1.2E-7	1.7E-3
mitochondrion	2.9E-20	7.8E-7	6.3E-4
mitochondrial part	2.9E-20	7.0E-6	1.8E-2
translational elongation	7.5E-20	4.7E-8	1.9E-4
cytosolic ribosome	4.4E-18	1.1E-7	8.0E-4
mitochondrial membrane part	1.1E-16	4.4E-6	-

The p-values for these GO terms are shown also for genes down-regulated only with moR-103a-2-3p mimic, and genes down-regulated only with miR-103a-3p mimic.

## Discussion

Canonical miRNAs form only a fraction of the small RNAs in many cell types. For example, sperm and oocytes contain abundant piwi-interacting RNAs (piRNAs) to protect genome integrity from the activity of retrotransposons [44], mRNA targeting endogenous siRNAs [45] derived for example from pseudogene-gene-transcript pairs, and the highly abundant tRNA-derived small RNA fragments (tRFs) [21,46–48]. For unknown reason, miRNA function is suppressed in mouse oocytes and early embryos [49,50] while the siRNA/piRNA fraction predominates, whereas during differentiation, the miRNA fraction increases, and siRNAs and piRNAs are few, or even lost [51]. Interestingly, pluripotent embryonic stem cells have been shown to express miRNAs, endo-siRNAs and a small fraction of piRNAs [13,14,21,51]. Also, up to ∼9% of the short reads in our hESC data matched tRNA sequences **(Fig. 1)** which suggests that also tRFs are highly abundant in hESCs. However, the diversity and significance of small RNA species other than miRNAs remain largely unknown. We sequenced small RNAs from in-house-derived hESC lines HS401 and HS181 and human foreskin fibroblast line HFF-1 to profile miRNAome and characterize for the first time the recently discovered miRNA relatives, microRNA-offset RNAs, with unknown function from these cells.

We discovered 350 unique microRNA-offset-like enrichments from the vicinity of 273 miRNA hairpins, which we refer to as moRNAs [21–27,37]. Several common characteristics with previously published reports emerged from our analysis. First, the identified moRNAs map precisely to the human genome and were located adjacent to the mature miRNA reads, in only a few cases overlapping the mature miRNA with one or two nucleotides. Second, like miRNAs, moRNAs were characterized by overlapping reads referred to as isomoRs. Third, the isomoR reads derived from the same 5’ arm of the miRNA hairpin were similar in their end, and isomoRs derived from 3’ arm were similar in their start. Fourth, the expression level of moRNAs was in most cases lower than the expression level of corresponding mature miRNA with few exceptions. Fifth, moRNAs derived most often from the vicinity of conserved miRNAs. Sixth, moRNA expression level did not correlate significantly with the expression of adjacent miRNA and abundant expression of an miRNA was not always accompanied with high expression of the adjacent moRNA, suggesting that the expression of these two related molecules is not necessarily interdependent. Further, in many hairpins, the prevalent moRNA was expressed from the arm of the minor miRNA as observed also in Gaffo et al., 2014 [37].

Interestingly, hESC data yielded the majority (326) of unique moRNAs while only 65 emerged from HFF-1 data. The difference is even higher in relation to the total amount of miRNA hairpin mapping reads which were notably fewer in hESCs than in HFF-1 sample. Out of 92 human moRNAs previously reported by Langenberger et al. 2009 [23] and 58 by Bortoluzzi et al. 2012 [26], we detected 58 and 37 overlapping sequences, respectively. Therefore, we report here the largest collection of unique moRNA reads derived from a human small RNA-seq experiment so far. In contrast to the almost exclusive detection of 5p moRNAs reported in both of the previous studies we found also abundant 3p forms in the hESC data. Duplex forming small RNA species such as miRNAs and tRFs are typically expressed in an asymmetric manner by favouring accumulation of either one strand derivative but not both, which indicates their non-random processing [52,53], and according to this and previous studies, moRNAs do not form an exception to this rule.

Because of the detection of 3p moRNAs in the hESC data, we were able to compare the 5p/3p moRNA lengths and their possibility to form a miRNA-like ∼20 nt duplex which appears at the miRNA maturation stage before their unfolding by effector complexes to target mRNA [54]. We observed a large variation in 3p moRNA lengths ranging from 16 to 29 nts when compared with relatively homogenous distribution of 5p moRNAs with a peak in 19 nt. Interestingly, similar length distribution was reported to tRFs and other terminal small RNA fragments which are processed from longer RNAs [47]. If opposing moRNAs form a short duplex, they would harbor various lengths of overhangs, distinct from 2 nt 3’ overhang processed by RNAse III enzymes Dicer or Drosha/DGCR8 [7,8]. The observation may indicate that, while the hairpin loop-side end of the moRNA may be determined by the Drosha/DGCR8 [22,23] the variable end probably results from alternative mechanisms. Recent reports have indeed shown an expression of a range of miRNA hairpin precursor variants with differing lengths and three dimensional structures which modulate the efficiency of miRNA processing [55–57]. Also relatively long, free 5’ overhangs [56] have been identified in some hairpins which may explain the accumulation of 5p moRNAs. However, 3’ overhangs longer than few nucleotides have not been reported so far. Therefore, molecular characterization of miRNA precursors, their binding enzymes and the resulting small RNA sequences will be essential for the elucidation of moRNA synthesis.

Most miRNAs deriving from the well-known pluripotency related miR-302/367, miR-371/372/373 and C19MC clusters were significantly overexpressed in our hESC data, a finding that is well in line with the earlier observations. Seven of the significantly differentially expressed moRNAs were derived from the hESC-specific miR-302/367 cluster. None of these sequences are found in other human moRNA studies [23,25,26], thus suggesting that in addition to miRNA expression, also the moRNA expression from this cluster may be specific to hESCs. Similarly, several moRNAs from the C19MC cluster are significantly overexpressed in the hESCs, but were not detected in earlier studies.

Before this study, human moRNAs have been reported only from neurons and cancer cells [23,25,26]. Also in our hESC data, many abundant moRNAs were derived from the miRNA clusters expressed in cancer. For example, moRNAs deriving from the c-myc induced miR-17-92 cluster [57] and the oncogenic miR-374b-421 cluster [58] have been detected also previously in the study by Bortoluzzi et al. 2012 [26]. On the other hand, these moRNAs were not present in our HFF-1 library. The moR-21-5p of the oncomir miR-21 was detected both in the HFF-1 library and by Bortoluzzi et al. 2012, while its opposing moR-21-3p expression was found only in our hESC data. Similarly, metastasis-associated mir-103a-2 cluster derived most abundant moRNA (moR-103a-2-5p) in Bortoluzzi et al. while the opposing moR-103a-2-3p was detected as second most abundant moRNA in hESCs. miR-103a-2 is not expressed by a consecutive hairpin cluster but as a bidirectional transcript from opposite DNA strands in chromosome 5, and it has also a bidirectional hairpin homolog (miR-103a-1) in chromosome 20. The homologous gene producing mir-103a-1 hairpin yielded also moRNA-like sequences, but in lesser extent both in our study and in the data of Bortoluzzi et al. 2012 [26]. miR-103a is shown to promote cancer-like properties by down-regulating KLF-4 and DAPK [38], but has also an interesting role in the modulation of miRNA processing and function via down-regulation of Dicer [39] and miRNA binding Argonaute AGO1 [59]. Interestingly, many moRNA-deriving, cancer-associated hairpins are also expressed in oocytes such as mir-17-92 cluster, miR-20, miR-21, miR-15a/16 and miR-103 [50] whereas miR-421 from mir-374b-421 cluster has been reported to be up-regulated in ovarian teratomas [60]. Taken together, moRNA expression seems to associate not only with certain cell types, but also with specific processes such as cancer and metastasis.

So far, no results concerning the possible functionality of moRNAs have been reported. In this study, we took up this challenge by transfecting HFF-1 cells with moR-103a-2-3p mimic and measured the effect with whole transcriptome microarrays. The set of genes down-regulated by moR-103a-2-3p was about as large as the set of genes down-regulated with the control, miR-103a-3p, suggesting a role for this moRNA in regulation of gene expression. However, it is unclear how moR-103a-2-3p affects gene expression; one possible way could be its direct binding to the target mRNAs 3’ UTR by the similar manner to canonical miRNAs. Consistent 5’ end of the sequence, a typical cleavage by RNA endonucleases such as Dicer and Drosha, suggests that it could have a role in target recognition in a 5’ seed based manner. Also, the observation, that moR-103a-2-3p 5’ seed matches were often found from 3’ UTRs of genes down-regulated by moR transfection but which instead were absent from the 3’ UTRs of genes that were down-regulated by miR transfection, may support the hypothesis. Even so, the approach taken in this study supports only the 3’ moRNAs, and dissection of functions of 5’ moRNAs with variable 5’ ends will require development of alternative approaches. While it is unsure if the observed changes in gene expression levels were caused by direct action of moR-103a-2-3p, also the mechanism how it induced the changes needs further investigation.

Notable fraction, 60%, of the genes down-regulated by the moR-mimic were down-regulated also by miR-mimic and were related to ribosomal or mitochondrial functions; these were also the most significant GO terms among the genes down-regulated only by moR-103a-2-3p. Instead, the genes down-regulated only by miR-103a-3p were associated also with other functions, part of which are formerly known. As a conclusion, it seems that moR-103a-2-3p would not have a function of its own, but could act as a co-player to miR-103a-3p and enhance its function in hESCs. We observed that the sequences of moR-103a-2-3p and miR-103-3p are partly similar which could explain some of the common regulated genes **(S2 Fig.).** The similarity is more prevalent at the latter half of the sequences (continuous at nucleotides 14–18) which excludes the possibility of canonical targeting of the same mRNA by the seed area. However, it could indicate either a novel recognition manner of the target mRNA, effector Argonaute competition situation, or attraction of Argonautes by similar sequence motifs. It is also possible that the absence of enriched GO terms uniquely related to moR-103a-2-3p is because the moRNA mimic is not expressed in the right cellular context or with suitable chemical modifications, as it is designed to imitate mature miRNA. Either, we cannot rule out the possibility that moR-103a-2-3p is a nonfunctional product from the miR-103a synthesis.

Small terminal RNAs have been recently reported emerging from most classes of longer RNAs such as tRNA (tRFs), rRNA, snoRNA and snRNA but not mRNA. Interestingly, moRNAs arise similarly as end fragments from pre-miRNAs and even exhibit similar length distributions to tRFs, 5' sequences being about 19nt and 3' sequences distributed broadly between 16nt-30nt, which may indicate a processing by common enzymatic machineries. Some of tRFs and snoRNA-derived small RNAs have been shown to function as miRNAs but also the effectiveness of miRNA loading to Argonautes has been shown to be modulated by some tRFs. It will be of interest to elucidate if moRNAs can take part to the Argonaute association-modulation.

## Materials and Methods

### Cell lines

Characterized, in house-derived hESC lines HS401 and HS181, and human primary foreskin fibroblast line HFF-1 (SCRC-1041) [2,61,62] were used to prepare small RNA-seq libraries to analyze their relative miRNA and moRNA expression profiles and to discover novel miRNA and moRNA sequences. Small RNA-seq data from hESC lines H9 and embryoid bodies (EB) published by Morin et al. 2008 [35], and from hESC line H1 and spontaneously differentiated cells published by Bar et al. 2008 [36] were analyzed only for moRNA sequences first detected in HS401, HS181 and HFF-1. hESC line FES29, uncharacterized iPS lines HEL23 and HEL41, and HEK293 were used only for qRT-PCR studies. Cells at the following passages (p) were used: HS401 p40 (miRNA-seq) and p42 (qRT-PCR), HS181 p63 (miRNA-seq) and p51 (qRT-PCR), HFF-1 p16 (miRNA-seq) and p4–16 (qRT-PCR), H9 p45-p55 (qRT-PCR), FES29 p31-p45 (qRT-PCR), HEL23 p5-p8 (qRT-PCR), HEL41 p5-p8 (qRT-PCR). Passage number of the cell line HEK293 was not determined.

### Cell culture and RNA isolation

For Small RNA-seq, hESC lines HS401 and HS181 were cultured on irradiated HFF-1 feeder layer in KnockOut Dulbecco's Modified Eagle Medium (DMEM) supplemented with Knockout Serum Replacement (Life Technologies Ltd, UK) and 8ng/ml basic fibroblast growth factor (FGF-2; R&D Systems, Minneapolis, MN, US), and passaged enzymatically in the presence of ROCK inhibitor Y-27632 (Calbiochem, Merck KGaA, Darmstadt, Germany) [63]. For small RNA-seq, the HFF-1 line was cultured in cell culture plates without coating substrates in Iscove's Modified Dulbecco's Medium (IMDM) supplemented with 10% fetal bovine serum (Life Technologies). For qRT-PCR, hESC lines HS401 and HS181 were cultured in similar conditions as for miRNA-seq, and hESC lines H9 and FES29 in feeder-free conditions on Matrigel and STEMPRO hESC SFM (Life Technologies) supplemented with 8ng/ml of FGF-2 (Life Technologies).

Cells were harvested using TryplE (Life Technologies) and total RNA was extracted using TRIzol (Life Technologies) according to the manufacturer’s protocol. The quality of total RNA was analyzed by the Nano 6000 (chip) Kit for Bioanalyzer (Agilent Technologies, Santa Clara, CA) and only samples with an RNA Integrity Number (RIN) greater than 9.0 were used for preparation of small RNA libraries.

### Small RNA-seq library preparation

Small RNA libraries were prepared from lines HS401, HS181 and HFF-1 using Illumina Small RNA Sample Prep-kit v 1.0 (Illumina Inc, San Diego, CA) as described earlier [64]. Ten μg of total RNA from each cell line was size fractionated using a 15% Novex gel (Life Technologies) and fractions corresponding to 15–40 nucleotides (nt) were excised for further preparation. The purified small RNA fraction was ligated into 5’- and 3’ end adapters, and the final product was reverse transcribed, PCR amplified 15 cycles, and sequenced with Genome analyzer IIX (Illumina). The raw data files are available at the Gene Expression Omnibus (GSE62501).

### Small RNA data analysis

After initial pre-processing by standard modules used with the Illumina Genome Analyzer IIX which include Firecrest for image analysis, Bustard for base calling and GERALD for the first genome alignment, the reads were preprocessed by removing bad quality reads (mean base quality Q<20) and low quality ends. Subsequently, 3’ adapters were trimmed using in-house tools, and adaptor dimers, homopolymers and too short reads (<14nt) were discarded. The trimmed reads were aligned to human genome hg19 using Bowtie 0.12.7 [65], allowing at most two mismatches and not more than 10 possible origins in the genome. Reads that aligned to the area of known human miRNA hairpins (miRBase v 17, April 2011, http://www.mirbase.org/) [66] were taken apart to create their own data set, and the number of reads in this set that aligned to annotated mature miRNA loci extended with two nucleotides both upstream and downstream was counted. Mature miRNAs which were detected with expression of at least 1 read per million mapped reads were included into the differential expression analysis between the hESC lines (HS401 and HS181) and HFF-1, which was performed using R/Bioconductor package DESeq [67]. DEseq makes the assumption of a negative binomial distribution and a locally linear relationship between over-dispersion and mean expression levels of the data. A miRNA was considered to be differentially expressed between hESC and HFF-1 libraries if the p-value was less than 0.0005. Because we did not have replicates of HFF-1 data, DESeq estimated the dispersion based only on the ES replicates. There are differences in gene expression between independently-derived hESC lines [68,69] and it has been shown that several pluripotency-related transcription factors are heterogeneously expressed in mouse ES cell lines [69–72]. One of those fluctuating TFs is NANOG [73], which is also involved in activation of ES cell miRNAs miR-290 and miR-302 [74]. Hence, we had a reason to believe that the variation of miRNA expression in hESCs is likely larger than the variation in HFF-1.

The reads that did not align to known miRNA hairpins were further searched for perfect matches with other small RNA sequences: snRNAs, snoRNAs, rRNAs, mitochondrial tRNAs, misc RNAs downloaded from ensembl (http://www.ensembl.org), piRNAs from piRNAbank [75] and Genbank (http://www.ncbi.nih.gov/genbank), tRNAs from UCSC (http://genome.ucsc.edu), and human genome repeats from Repbase September 2011 release [76]. The reads that could be aligned to distinct small RNA classes were filtered out from the data; the read count information during the filtering steps is shown in **S1 Table**.

miRNA hairpin precursors associated to only one known mature miRNA product were searched for new minor 5p or 3p miRNA forms requiring that a candidate sequence has at least 10 occurrences, maps with no mismatches to the arm opposite of the known mature miRNA, and has strong base pairing (≥14 bp within the first 20 nt) with the known mature miRNA. Further, in order to find novel miRNAs, the reads not mapped to any small RNA species were studied with miRDeep2 [77]. Further criteria used to consider a predicted miRNA hairpin as a miRNA candidate were: (1) at least 10 exactly mapping reads for the main miRNA product of the hairpin, (2) at most 10 genomic copies, and (3) GC content in known miRNA range (15–90%). We divided the novel miRNA hairpins into conserved and nonconserved ones using the PhastCons data [78] available in UCSC (http://genome.ucsc.edu). PhastCons values show the probability that a nucleotide belongs to a conserved element, and here the limit for a hairpin to be conserved was set to mean PhastCons value 0.5 (conserved in mammals).

Candidate moRNAs were searched from the vicinity of known miRNA precursors. First, reads that mapped to the area of miRNA hairpin sequences (miRBase release 17) extended with 30 nt both upstream and downstream were gathered, and reads that mapped to the miRNA hairpin sequence area were excluded from this set. We considered those reads located at 5’ side of miRNA hairpin as putative 5’ moRNA sequences. Similarly, reads that mapped to 3’ side area of the hairpin were considered as putative 3’ moRNA sequences. Only reads mapping to the same strand with the miRNA precursor were counted as possible moRNAs. The differentially expressed moRNAs between ES and fibroblast libraries were searched the same way as the differentially expressed miRNAs. Due to large variability in unique moRNAs with low read counts and detection of common moRNAs with high read counts in hESC samples, shown also by moRNA relative expression analysis, only sequences with at least 5 reads per sample and 0.5 reads per million (RPM) were taken to size distribution analysis.

### Small RNA qRT-PCR

Total RNA was extracted for qRT-PCR with miRVana miRNA isolation-kit (Life Technologies) and reverse transcribed with TaqMan Reverse Transcription-kit (Life Technologies). Small RNA-specific TaqMan assays (common catalog number: 4427976) were purchased from Life Technologies for hsa-miR-302a-3p (000529), hsa-miR-302d-3p (000535), hsa-miR-372 (000560), hsa-let7g-5p (002282), miR-145-5p (002278), miR-103-3p (000439), miR-103-5p (121218_mat). Custom TaqMan small RNA assays were purchased for moR-103-3p (CSHSNOI), moR-103-5p (CS1LUQ), moR-367-3p (CSFARB2) and moR-367-5p (CSGJPIA). RNU44 (001094) was used as an endogenous control to quantify pre-miR-103a-2 derivatives (two miRs and two moRs) in **Fig. 2 C**. RNU6B (001093) was used as an endogenous control to quantify hESC/HFF-1 miRNAs in **S1 Fig.** 7.5ng of cDNA per 15μl reaction was run with TaqMan Universal Master Mix II (Life Technologies) in Rotor Gene 6000 (Qiagen/Corbett Research, Australia). All experiments were run in three technical and three additional biological replicates unless otherwise stated.

### Transfection of small RNA mimics

miRVana small RNA mimics were purchased from Life Technologies. Sixty nM of custom design moR-103-3p (AAGAACCAAGAAUGGGCUGC), miR-103-3p (4464066) or negative control #1 (neg#1, 4464058) was transfected twice at following days using 1.5 μl/ml RNAi max reagent (Life Technologies) starting from approximately 40% confluent HFF-1 cells in serum-free KnockOut DMEM. Three independent biological samples were prepared for three conditions: moR-103-3p, miR-103-3p, neg#1.

### Microarray and Gene Ontology analysis

Human HT12 V4 whole transcript arrays (Illumina) were hybridized in Biomedicum Helsinki Functional Genomics Unit (FUGU). Microarray data analysis was performed using GeneSpring, http://www.genomics.agilent.com. Correlation coefficients and Principal Component Analysis were used to asses sample quality. Poor quality signals were removed by filtering by percentile (lower cut-off 10% and upper cut-off 98%). Benjamini Hochberg False Discovery Rate corrected t-test was applied to identify differentially expressed genes between (two) conditions. DAVID Bioinformatics database [43] was used for functional annotation clustering and Gene Ontology analysis, http://david.abcc.ncifcrf.gov/home.jsp. Modified t-test, referred as EASE score, was applied to calculate p-value of enriched Gene Ontology categories.

## Supporting Information

S1 Table Number of reads mapping to distinct classes of ncRNAs. (XLSX)

S2 Table miRNA differential expression. (XLSX)

S3 Table moRNA summary. (XLSX)

S4 Table moRNA differential expression. (XLSX)

S5 Table Differentially expressed genes after transfections. (XLSX)

S6 Table Enriched Gene Ontologies. (XLSX)

S7 Table Predicted targets. (XLSX)

S1 Fig miRNA qPCR. (DOCX)

S2 Fig ClustalW analysis of miR vs. moR sequences. (PPTX)
