# Supplementary material for: Selective MicroRNA-Offset RNA Expression in Human Embryonic Stem Cells
Source: PLoS One. 2015 Mar 30;10(3):e0116668. doi: 10.1371/journal.pone.0116668 (PMC4378994; doi:10.1371/journal.pone.0116668)

**Supplementary Figure S1.** Relative expression of hESC-specific miRNAs (miR-302a-3p/d-3p and miR-372) and differentiation driving miRNAs (let-7g-5p, miR-145-5p) in HS181 and HS401 against HFF-1 fibroblast line. If the signal was absent, ct value 40 was used to calculate relative expression. n=3.


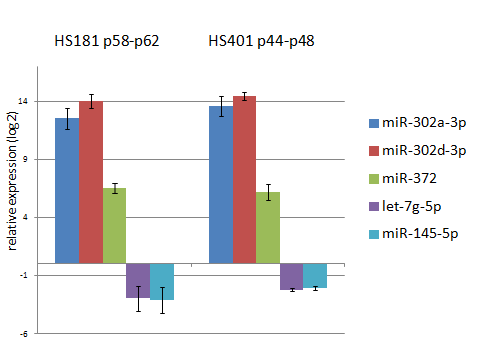

Supplement: S1 Fig — (DOCX) [file pone.0116668.s008.docx]
