# Supplementary figures and images for: Selective MicroRNA-Offset RNA Expression in Human Embryonic Stem Cells
Source: PLoS One. 2015 Mar 30;10(3):e0116668. doi: 10.1371/journal.pone.0116668 (PMC4378994; doi:10.1371/journal.pone.0116668)

## Slide 1
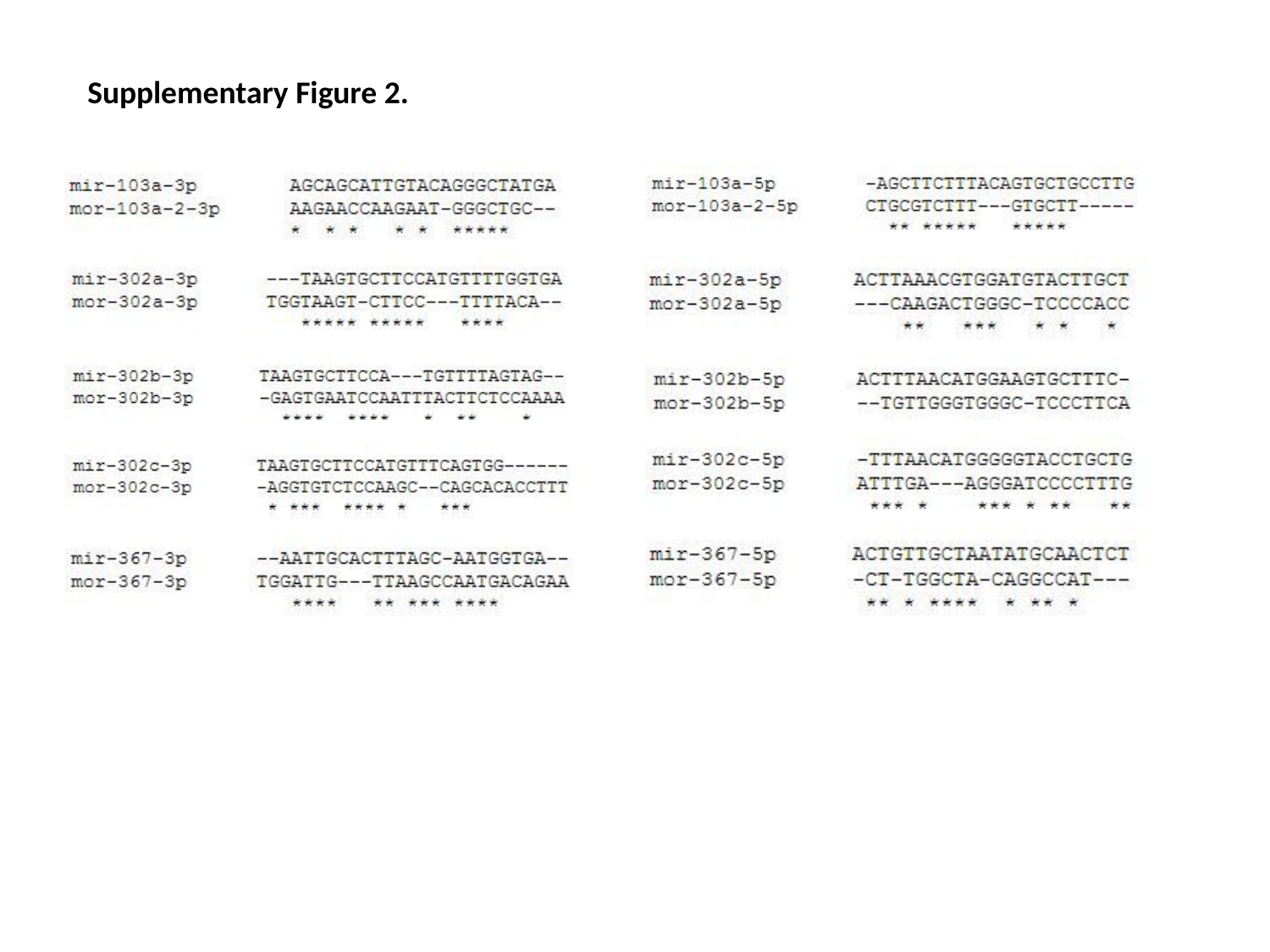

Supplementary Figure 2.

Supplement: S2 Fig — (PPTX) [file pone.0116668.s009.pptx]
